# G2S: A New Deep Learning Tool for Predicting Stool Microbiome Structure From Oral Microbiome Data

**DOI:** 10.3389/fgene.2021.644516

**Published:** 2021-04-09

**Authors:** Simone Rampelli, Marco Fabbrini, Marco Candela, Elena Biagi, Patrizia Brigidi, Silvia Turroni

**Affiliations:** ^1^Unit of Microbiome Science and Biotechnology, Department of Pharmacy and Biotechnology, University of Bologna, Bologna, Italy; ^2^Department of Medical and Surgical Sciences, University of Bologna, Bologna, Italy

**Keywords:** gut microbiome, oral microbiome, deep learning, microbiome, paleomicrobiology

## Abstract

Deep learning methodologies have revolutionized prediction in many fields and show the potential to do the same in microbial metagenomics. However, deep learning is still unexplored in the field of microbiology, with only a few software designed to work with microbiome data. Within the meta-community theory, we foresee new perspectives for the development and application of deep learning algorithms in the field of the human microbiome. In this context, we developed G2S, a bioinformatic tool for taxonomic prediction of the human fecal microbiome directly from the oral microbiome data of the same individual. The tool uses a deep convolutional neural network trained on paired oral and fecal samples from populations across the globe, which allows inferring the stool microbiome at the family level more accurately than other available approaches. The tool can be used in retrospective studies, where fecal sampling was not performed, and especially in the field of paleomicrobiology, as a unique opportunity to recover data related to ancient gut microbiome configurations. G2S was validated on already characterized oral and fecal sample pairs, and then applied to ancient microbiome data from dental calculi, to derive putative intestinal components in medieval subjects.

## Introduction

Deep learning is increasingly being used to make inference on large and complex data. Unlike traditional algorithms, in which the expertise and rules are already coded, deep learning algorithms are built to automatically detect patterns in data (Murphy, 2012; Bishop, 2016), also embedding the computation of variables into the models themselves to yield end-to-end models (Goodfellow et al., 2016). In particular, the construction and training of deep learning algorithms have been enabled by the increasing availability of big data and the rapid growth in the number and size of public available databases. So far, deep neural networks have been key to advances in modern artificial intelligence, with applications such as facial recognition, speech recognition and self-driving vehicles. More recently, new applications have been pioneered in the fields of molecular biology and metagenomics. Indeed, the same deep learning approaches are beginning to be applied to genetics, agriculture and medicine (Alipanahi et al., 2015; Leung et al., 2016; Ching et al., 2018; Demirci et al., 2018; Wainberg et al., 2018; Webb, 2018; Le, 2019; Le and Huynh, 2019; Le et al., 2019; Quang and Xie, 2019). However, deep learning is still unexplored in the field of microbial metagenomics, with only a few approaches suitable for microbiome data (Geman et al., 2016; Reiman et al., 2017; Galkin et al., 2020), and a huge untapped potential yet unexplored.

The human microbiome, i.e., the sum of the different microbial ecosystems that colonize the niches of the human body, plays an important role in human physiology and its dysbiotic variations can severely impact our health (Kau et al., 2011). For example, shifts in the composition of microbial communities inhabiting the oral cavity and gastrointestinal tract have been associated with the onset and/or progression of various conditions, such as periodontitis (Griffen et al., 2012) and other modern chronic disorders, including inflammatory bowel disease (Glassner et al., 2020), obesity (Rampelli et al., 2018), cardiovascular disease (Pietiäinen et al., 2018) and some forms of cancer (Helmink et al., 2019; Karpiński, 2019; Wong and Yu, 2019). The importance of the human microbiome in health and disease makes it imperative to understand the drivers of its variation. In this context, a new frontier is represented by the meta-community theory, according to which human symbiont microbial ecosystems are in intimate connection, showing reciprocal influences and exchanges (Koskella et al., 2017; Miller et al., 2018). Supporting a meta-community view of human microbial ecology, a close link between oral and intestinal microbiomes has recently been hypothesized, with the former reflecting changes in the latter, in both healthy and diseased individuals (Bajaj et al., 2015; Iwauchi et al., 2019; Prodan et al., 2019; Schmidt et al., 2019). Another scale of human microbiome variation is represented by its change across the evolutionary timeline. In particular, a large body of literature indicates that the current human gut microbiome has evolved toward at least two different configurations, rural and urban, both associated with the corresponding subsistence strategy. Compared to the first, generally considered as the pristine human gut microbiome, the urban configuration is characterized by an overall compression of microbial biodiversity, a wholescale loss of commensal microbial groups, and an increased presence of genes related to antibiotic resistance and xenobiotics metabolism (Yatsunenko et al., 2012; Schnorr et al., 2014; Obregon-Tito et al., 2015; Rampelli et al., 2015; Ayeni et al., 2018; Jha et al., 2018). These changes, collectively referred to as “microbiota insufficiency syndrome” (Sonnenburg and Sonnenburg, 2019), have been identified as contributing factors to the rise in chronic inflammatory non-communicable diseases. However, mainly due to the paucity of ancient stool samples, the truly ancestral human gut microbiome is still unknown and the evolutionary trajectories and drivers leading to its contemporary configurations have yet to be described, leaving important gaps in knowledge of the gut microbiome-human host co-evolutionary trajectories. Contrary to ancient fecal samples, dental ones are more common and well preserved, allowing for the extraction of the ancient oral microbiome from ancient DNA preserved in dental tartar. Consistent with the meta-community vision, the ancient configuration of the oral microbiome can somehow mirror the structural features of the intestinal one due to the intrinsic connections between the two ecosystems. In this scenario, here we developed a new deep learning-based tool, G2S, which infers the gut microbiome configuration from the oral microbiome data of a given individual. G2S is based on a model trained and tested on a total of 305 and 79 paired samples of oral and stool microbiome, respectively, retrieved from multiple studies with individuals of various geographical origins, including United States, Fiji, United Kingdom, and European countries (The Human Microbiome Project Consortium, 2012; Zaura et al., 2015; Brito et al., 2016; Russo et al., 2018). Our approach may be relevant for predicting the gut microbiome configuration when fecal data are not available, and particularly suitable for human archeological records, where coprolites and fecal sediments are indeed rare compared to dental calculi and other human remains.

## Materials and Methods

G2S software is built in an R environment, using the R packages “base,” “stats,” and “keras,” containing “tensorflow.” The G2S source code is available on the website https://github.com/simonerampelli/g2s and it can be run using a command line interface on computer with Windows, Linux and OS X as the operating system.

The G2S tool was trained and tested on a total of 768 paired samples (i.e., oral and stool samples from the same 384 individuals), including samples from 171 healthy adults from United States, 7 from Italy, 29 from Sweden, 37 from United Kingdom, and 140 from Fiji (The Human Microbiome Project Consortium, 2012; Zaura et al., 2015; Brito et al., 2016; Russo et al., 2018). Eighty% of the subjects were used for the training dataset and 20% for the test dataset, without overlapping to avoid overfitting. Both 16S rRNA gene reads and shotgun metagenomics sequences were used, analyzed by the QIIME 2 pipeline (Bolyen et al., 2019) or the MetaPhlAn2 software (Truong et al., 2015), respectively.

The performance of G2S in predicting fecal microbiome configuration from the same individual’s oral microbiome sample was compared with that of other available approaches, including Random Forest (Breiman, 2001) and a stochastic algorithm, i.e., a customized method that generates mock profiles of the stool microbiome by randomly imputing the abundances of bacterial families in the range of the training dataset (see Supplementary File 1 for script source).

Microbiome data from dental calculi of 4 adult human skeletons (G12, B17, B61, and B78), characterized by sequencing the V5 and V6 regions of the 16S rRNA gene (8 samples in total) (Warinner et al., 2014), were used to illustrate the potential and results of G2S. No ethics committee approval was required to perform the analysis included in this study.

## Results

### Implementation of the G2S Software

G2S adapted a deep convolutional neural network (ConvNet) to predict gut microbiome configurations from oral microbiome data. Several model architectures were tested in order to find the best performing algorithm, either by testing hidden layers with different number of units, and/or by adding a weight regularization step or a dropout procedure (data not shown). The final ConvNet was structured with two hidden layers, each with 50 units, and a final linear layer with 13 units and no activation function. We selected mean square error as the loss function, and mean absolute error as the metric to evaluate the differences between predictions and targets during training. In order to minimize overfitting problems due to the small number of samples within the dataset, we also included a weight regularization step, by adding to the loss function a cost associated with having high weights. The cost was proportional to the square of the weight coefficient value (L2 regularization or weight decay). Finally, to further prevent overfitting, dropout was applied to the first two layers, obtaining a better prediction and a significant reduction in losses and minimum absolute errors with a rate value of 0.5.

**FIGURE 1:**
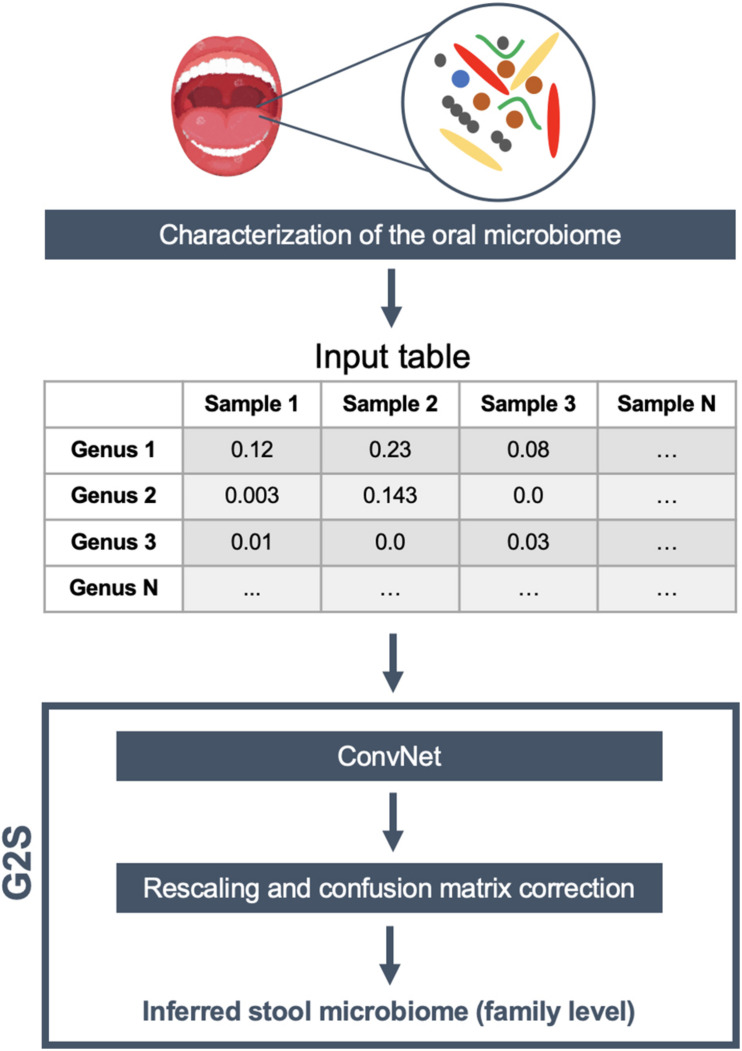
G2S workflow. The input file is a genus-level relative abundance table (.tsv format), obtained from the characterization of human oral microbiome samples. The stool microbiome is inferred using a deep convolutional neural network (ConvNet) adjusted by a confusion matrix and rescaled to 100%. The results are tabulated as relative abundance.

For ConvNet training and testing, we downloaded all available paired samples (i.e., gingival and stool samples from the same individual) from the HMP project (The Human Microbiome Project Consortium, 2012). In order to increase the generalization capability of our ConvNets, while minimizing geography-related bias (He et al., 2018), we integrated our dataset with all available paired samples (i.e., oral and fecal samples) from healthy adults from other literature studies (Zaura et al., 2015*;* Brito et al., 2016*;* Russo et al., 2018), selecting both 16S rRNA gene and shotgun metagenomic datasets (see also Supplementary Table 1). Our final dataset included paired samples of 171 individuals from United States, 7 from Italy, 29 from Sweden, 37 from United Kingdom, and 140 from Fiji, for a total of 384 oral and 384 stool samples, divided into 528 16S rRNA gene and 240 shotgun fastq files. Specifically, 16S rRNA gene sequences were analyzed using the QIIME 2 pipeline (Bolyen et al., 2019) and the Greengenes database (DeSantis et al., 2006) in order to obtain the microbiome classification at different taxonomic levels. On the other hand, the shotgun metagenomic samples were analyzed by MetaPhlAn2 (Truong et al., 2015) using the default parameters. The genus-level abundance table of 384 oral microbiome samples was normalized feature-wise prior to its usage for deep learning. In particular, the data were centered on the mean of each specific genus and scaled according to their standard deviation. Only 50 genera present in more than 4 samples with relative abundance greater than 0.1% were retained for the analysis. The 12 bacterial families of the stool microbiome dataset with the highest contribution in terms of median relative abundance, including *Bacteroidaceae, Porphyromonadaceae, Lachnospiraceae, Ruminococcaceae, Veillonellaceae, Rikenellaceae, Alcaligenaceae, Streptococcaceae, Bifidobacteriaceae, Clostridiaceae, Prevotellaceae*, and *Erysipelotrichaceae*, were selected as features to be predicted by ConvNet analysis. An additional variable, called “Other” (i.e., the percentage remaining to reach 100%), was also considered a feature to be inferred. The training and test datasets were separated to contain 80 and 20% of all profiles, i.e., 305 and 79 paired oral and fecal samples, respectively. In order to better evaluate the model, we used a k-fold cross-validation approach with 4 partitions and 500 epochs. We got the best performance after the 151st epoch, with a mean absolute error of 4.1%. To increase the predictive performance of ConvNet, the results were then transformed as follows: (i) negative predictions were set to 0, and (ii) the sum of the value for each sample was rescaled to 100%. Finally, based on the results of the training dataset, we also built a confusion matrix to adjust the predictions of those families with recurring over- or underestimation. G2S includes all of these steps in a single R script, and requires only a relative abundance table of the oral microbiome (between 0 and 1) at the genus level with samples in the columns and the full taxonomy following the Greengenes_05_2013 style in the rows as input file. For each sample analyzed, the predicted microbiome is summarized in a table as the relative abundance of the most abundant bacterial families. Additionally, histograms of the same families are provided, using the “graphics” and “base” R packages. The schematic overview of the G2S framework is provided in Figure 1.

### Ascertaining the Performance of G2S on the Test Dataset

**FIGURE 2:**
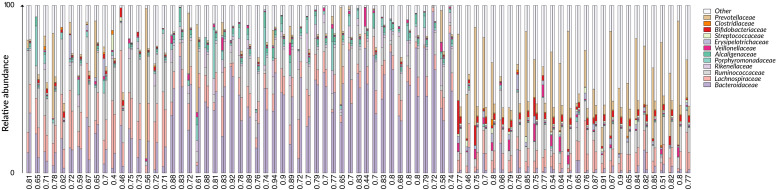
Comparison between G2S predictions and real data from the test dataset. The family level bar plots of the 79 stool samples of the test dataset are visualized next to their inferred configurations obtained by G2S. Spearman correlation coefficients (r) are provided below each pair of bar plots. Samples are derived from the following studies: The Human Microbiome Project Consortium (2012), Zaura et al. (2015); Brito et al. (2016), Russo et al. (2018).

**FIGURE 3:**
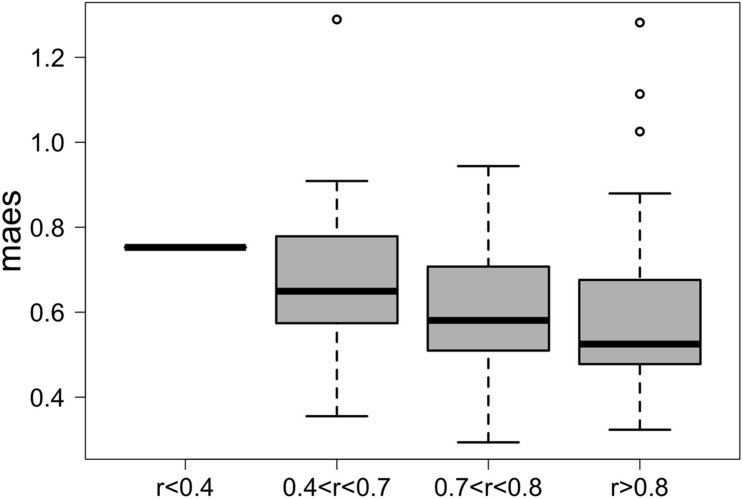
G2S predictions are more accurate when the configurations to be inferred fall within the plane of variation of the training dataset. Box plots of the mean absolute error scaled to one standard deviation (maes) between the real stool microbiome configuration of the samples in the test dataset and the median configuration of the training dataset. Samples were divided into four groups based on the quality of the G2S predictions (i.e., the Spearman correlation coefficients between the real values and the inferred configurations).

We first applied G2S to the test dataset to evaluate its cross-validated predictions. In particular, mean absolute errors for each family scaled to one standard deviation of real data (maes) < 1 were considered as reference parameters for a good quality of the prediction. As expected, G2S predicts relative abundances with an average maes of 0.59, ranging from the best score for *Bacteroidaceae* and *Erysipelotrichaceae* (maes = 0.46) to the worst case for *Ruminococcaceae* (maes = 0.77). To gain more insights into the predictive performance of G2S, we globally compared, sample by sample, the inferred microbiome configurations with real data by means of bar plots (Figure 2). Spearman correlations between predicted and actual microbiome profiles were used to evaluate predictions for each subject. In particular, we considered as excellent those predictions with r > 0.8 (52% of predictions), good those with r between 0.71 and 0.8 (29% of predictions), discrete with *r* between 0.41 and 0.7 (18% of predictions), and incorrect with *r* ≤ 0.4 (1% of predictions). When we analyzed the single case in which G2S inferred an incorrect prediction, we found that the stool microbiome configuration was very peculiar, with the relative abundances of the two keystone bacterial families *Bacteroidaceae* and *Lachnospiraceae* not reaching 5% of relative abundance together (while generally dominant in the ecosystem). It is important to note that G2S worked correctly even when the stool microbiome configurations to be predicted were not so close to the median configuration of the training dataset (maes < 1 even when *r* < 0.7) (Figure 3). This was likely due to the large variation captured by the pool of microbiome configurations of the samples in the training dataset.

**FIGURE 4:**
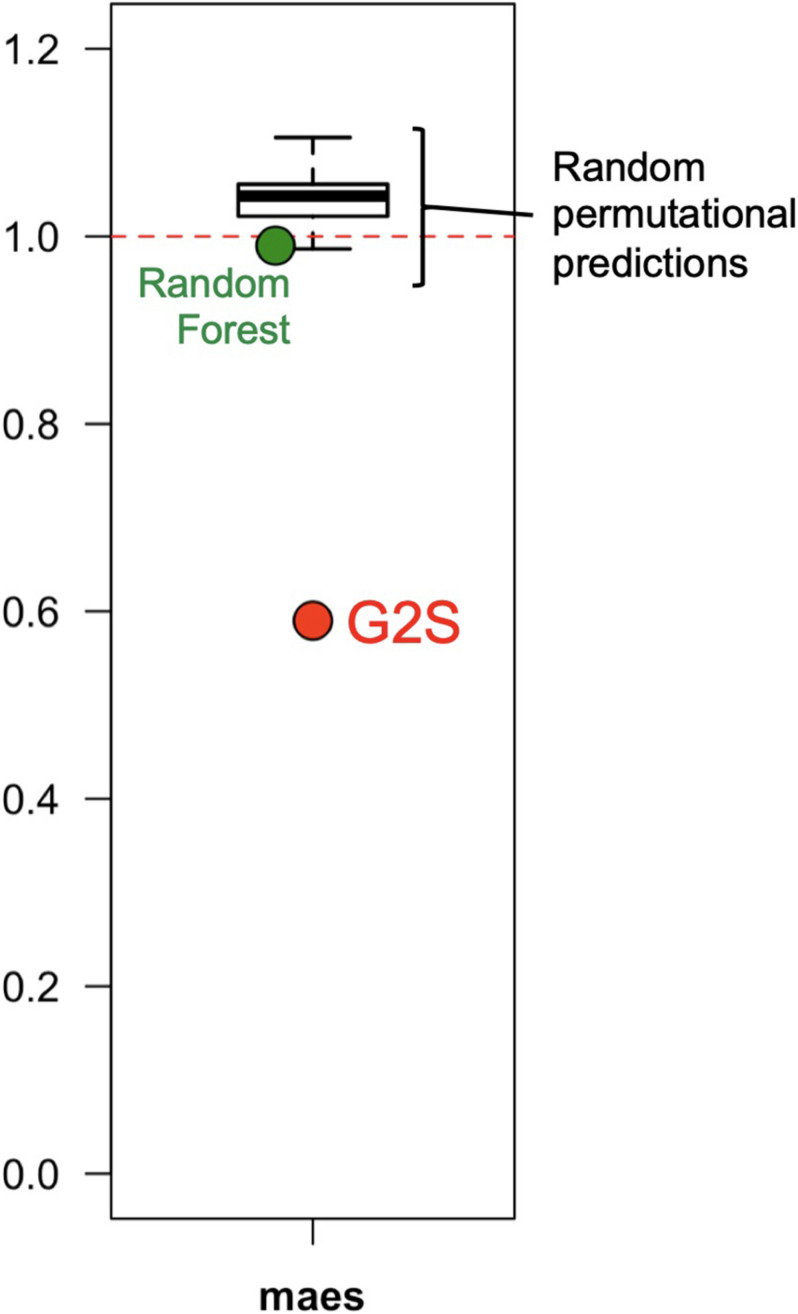
G2S predicts the stool microbiome configuration with better performance than other methods. The mean absolute errors scaled to one standard deviation (maes) between the real data of the samples from the test dataset and the configurations inferred by G2S, Random Forest and a stochastic permutational method (100 predictions), are reported in the dot plot.

G2S showed a better mimicry of the relative abundance of microbiomes in the test dataset than other methods, including Random Forest and a stochastic method developed specifically for this comparison, which generates mock profiles of the stool microbiome in the range of the training dataset (Figure 4). Random Forest under- or overestimated bacterial families with a global maes of 0.99, ranging from 0.77 for *Bacteroidaceae* to 1.74 for *Streptococcaceae*. The performance of our custom predictor was even more inaccurate, with a total of 100 permutational predictions showing maes between 0.98 and 1.11 (mean = 1.05). The best performance of G2S in predicting the stool microbiome structure is probably due to the predictive power of deep learning that automatically detects patterns in the data, by also embedding the computation of variables into the models themselves to yield end-to-end models.

### Case Study: Using G2S in Paleomicrobiology to Predict the Stool Microbiome Profile From Ancient Dental Calculi

**FIGURE 5:**
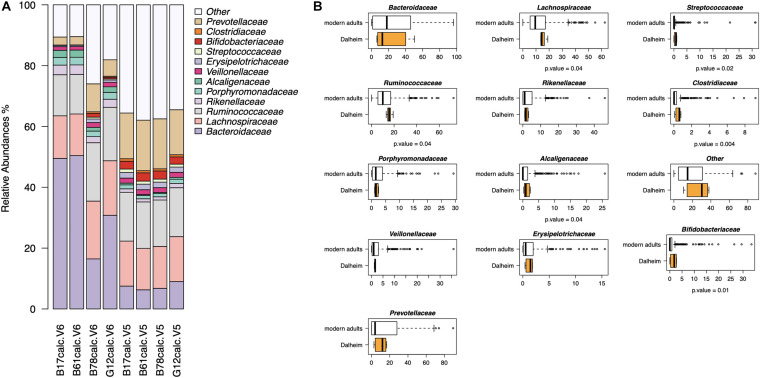
Reconstructing the ancient stool microbiome of adult medieval individuals. **(A)** Bar plots of stool microbiome configurations inferred from 16S rRNA gene (V5 and V6 regions) sequencing data of ancient microbiomes (i.e., dental calculi from the medieval monastic site of Dalheim, Germany [ca. 950–1,200 CE]) (Warinner et al., 2014). **(B)** Comparison between the predicted ancient microbiome configurations and the modern stool microbiome of subjects from the dataset used to implement G2S (The Human Microbiome Project Consortium, 2012; Zaura et al., 2015; Brito et al., 2016; Russo et al., 2018). *P*-values were determined by Wilcoxon test.

In the second part of our analysis, we used G2S to infer the stool microbiome from oral microbiome data of four adult human skeletons with evidence of mild to severe periodontal disease, from the medieval monastic site of Dalheim, Germany (ca. 950–1,200 CE) (Warinner et al., 2014). G2S inferred the stool microbiome structure at the family level, estimating the abundance of the 13 features, i.e., the 12 bacterial families and the category “Other” including all other families (Figure 5A). Interestingly, *Bacteroidaceae, Lachnospiraceae, Ruminococcaceae*, and *Prevotellaceae* were the predicted dominant components in the feces of the four subjects, using both V5 and V6 regions as targets of the 16S rRNA gene (together their relative abundance ranged from 52 to 80%). On the other hand, the family *Clostridiaceae* showed the lowest relative abundance (<1%) in all eight samples. Significant differences in taxon relative abundance were found with respect to the stool microbiome of modern subjects from the dataset used to implement G2S, including higher relative abundance of *Ruminococcaceae*, *Lachnospiraceae, Streptococcaceae, Alcaligenaceae, Clostridiaceae*, and *Bifidobacteriaceae* in the predicted ancient microbiome configurations (*p*-value < 0.05, Wilcoxon test) (Figure 5B). This is not unexpected given the profoundly different lifestyles of ancient individuals of the Middle Ages and modern people, in terms of diet, contact with the environment and sanitization practices (The Human Microbiome Project Consortium, 2012; Warinner et al., 2014; Zaura et al., 2015; Brito et al., 2016; Russo et al., 2018). Future studies in larger worldwide cohorts, including paired samples of oral and intestinal microbiome, are needed to refine the accuracy of the G2S software and predict a higher number of bacterial families as well as possibly taxa at different phylogenetic levels, possibly including genera and species.

## Discussion

G2S is specifically designed to predict the structure of the human stool microbiome from oral microbiome data. In particular, it uses relative abundance tables of the oral microbiome generated by next-generation sequencing, and a deep learning approach that allows high-speed prediction of the stool microbiome without any downstream process. It could be used with both modern and ancient samples, providing a good prediction of the fecal microbiome with a net saving of time and costs. This is particularly relevant in the context of paleomicrobiology, where human coprolites and fecal sediments are very rare compared to dental calculi. However, as G2S appears to work best when the input oral microbial composition is close to the average used during training, caution must still be taken in interpreting the prediction data. Furthermore, G2S was implemented using both 16S rRNA gene and shotgun metagenomics data from different populations across the globe (from United States, Italy, Sweden, United Kingdom, and Fiji), with a good generalization of the results as evidenced by the findings on the test dataset. This provides an opportunity for users who can apply the tool on data obtained through different sequencing techniques simply by formatting their abundance tables with a taxonomy congruent with the Greengenes database. It should also be noted that G2S was built and validated using the 768 paired samples currently available in the literature. This stresses the importance of collecting paired samples (i.e., oral and fecal) in future studies from cohorts from different geographic locations, in order to further extend the range of the training dataset and thus the applicability of G2S. Finally, other future implementations could include predictions at different taxonomic levels, as well as functional predictions thanks to the recent expansion of shotgun metagenomics.

In summary, G2S opens up new possibilities in bioinformatics approaches related to metagenomics, extending *in silico* procedures to predict the human stool microbiome from oral microbiome data. Starting from either modern or ancient oral microbiome samples, the tool infers the stool microbiome with family level resolution. Its main field of application is probably paleomicrobiology, as a tool that can help understand how the gut microbiome of the past was structured, and its implications for human evolution. An update of the G2S tool will be periodically performed to incorporate newly released microbiome studies.

## Data Availability Statement

The datasets used for setting up G2S are available at the Human Microbiome Project website https://www.hmpdacc.org/HMQCP/ and NCBI SRA as SRP057504 (Zaura et al., 2015), PRJNA217052 (Brito et al., 2016) and PRJNA356414 (Russo et al., 2018). Microbiome data from ancient samples were taken from the study conducted by Warinner and colleagues (Warinner et al., 2014).

## Author Contributions

SR: conceptualization and software. SR and MF: formal analysis. SR, MC, and ST: writing—original draft preparation. MF, EB, and PB: writing—review and editing. All authors have read and agreed to the published version of the manuscript.

## Conflict of Interest

The authors declare that the research was conducted in the absence of any commercial or financial relationships that could be construed as a potential conflict of interest.

## Supplementary Material

Supplementary File 1 R script containing the stochastic method that generates mock profiles of the stool microbiome in the range of the training dataset.

Supplementary Table 1 List of paired fecal and oral samples from the HMP study as well as from other literature studies dealing with healthy adults (Zaura et al., 2015; Brito et al., 2016; Russo et al., 2018). Both 16S rRNA gene sequencing and shotgun metagenomics studies were considered. For each sample, the following data are reported: sample ID, subject ID (and visit when available), geographical origin, reference, sequencing method and body site.
