# Supplementary material for: G2S: A New Deep Learning Tool for Predicting Stool Microbiome Structure From Oral Microbiome Data
Source: Front Genet. 2021 Apr 9;12:644516. doi: 10.3389/fgene.2021.644516 (PMC8062976; doi:10.3389/fgene.2021.644516)
Supplement: Supplementary File 1 — R script containing the stochastic method that generates mock profiles of the stool microbiome in the range of the training dataset. [file Presentation_1.pdf]

```
minimo=c(0,1.1154011, 0.6435006,0,0,0,0,0,0,0,0,0,0.2204586)
massimo=c(96.296296, 44.776821, 48.649649, 17.775612, 29.326531, 9.157936, 11.250189,
14.086919,  9.452736, 19.883050,  3.780110, 89.639954, 72.781750)
sd_training_set=c(25.9943781,  9.9005995, 10.6426553,  4.3898643,  5.3240909,  2.2173535,
2.5984619,  2.3853532, 1.3633424,  2.9297245,  0.6092083, 23.2080380, 17.0060593)

random=matrix(ncol=13,nrow=79)
perf_random=matrix(ncol=13,nrow=100)

for (z in 1:100)
{
 for (i in 1:79)
 {
  for (j in 1:13)
  {
   random[i,j]=runif(1,min=minimo[j],max=massimo[j])
  }
 }
 totale=rowSums(random)
 random1=random/totale*100
 perf_random[z,] <- colMeans(abs(random1-sales))/sd_training_set
}

colMeans(perf_random)
rowMeans(perf_random)

mean(rowMeans(perf_random))
```
